# Prevalence and Patterns of Congenital Coronary Artery Anomalies in Patients Undergoing Coronary Angiography at a Tertiary Care Hospital in the United Arab Emirates: A Retrospective Analysis

**DOI:** 10.7759/cureus.87576

**Published:** 2025-07-09

**Authors:** Aamer Zeen Al-Deen, Haitham Al-Hashimi, Basel Baaj, Kasem Siyagha, Salah Aldeen Roqia, Tasneem Fatima, Sadeq Tabatabai

**Affiliations:** 1 Graduate Medical Education Department, Mohammed Bin Rashid University of Medicine and Health Sciences, Dubai, ARE; 2 Cardiology Department, Rashid Hospital, Dubai Health, Dubai, ARE; 3 Internal Medicine Department, Dubai Hospital, Dubai Health, Dubai, ARE; 4 Cardiology Department, Dubai Hospital, Dubai Health, Dubai, ARE

**Keywords:** acute myocardial infarction, coronary artery angiography, coronary artery anomalies, coronary artery fistula, coronary origin anomalies, dubai, myocardial bridging (mb), prevalence, united arab emirates (uae)

## Abstract

Background

Coronary artery anomalies (CAAs) are uncommon congenital variations with potentially significant clinical implications, including myocardial ischemia and sudden cardiac death. Data on their prevalence and patterns in the United Arab Emirates (UAE), particularly in Dubai, remain limited.

Objectives

This study aimed to evaluate the prevalence, anatomical types, and clinical presentation of CAAs among patients undergoing coronary angiography at a tertiary care center in Dubai.

Methods

A retrospective analysis was conducted on all coronary angiograms performed at Rashid Hospital between January 1, 2017, and December 31, 2023. Two independent cardiologists reviewed the angiograms to identify CAAs. Clinical data, including presentation and left ventricular ejection fraction (LVEF), were extracted from electronic medical records.

Results

Among 6,228 coronary angiograms, 92 patients (1.48%) were found to have CAAs. Myocardial bridging was the most frequent anomaly (52.2%), followed by anomalies of coronary origin (45.7%) and coronary artery fistula (2.2%). The majority of patients were men (85.9%), with a mean age of 54.0 ± 11.5 years. Acute myocardial infarction (AMI) was the most common presentation (63.0%). Most patients had preserved left ventricular ejection fraction (67%) and were discharged alive (97.8%). AMI occurred more frequently in origin-related CAAs (73.8%), while anginal symptoms were more common in course-related CAAs (37.5% versus 14.3%, P = 0.086).

Conclusion

CAAs were infrequent but clinically relevant findings in patients undergoing coronary angiography in Dubai. Myocardial bridging was the predominant anomaly. These results underscore the importance of systematic evaluation for CAAs during angiographic assessment. Further multicenter research utilizing advanced imaging and long-term follow-up is needed to refine risk stratification and management strategies.

## Introduction

Coronary artery anomalies (CAAs) are uncommon yet clinically significant congenital variations in coronary anatomy. They comprise a heterogeneous group of structural abnormalities involving the anomalies of the origin and course, anomalies of intrinsic anatomy, anomalies of termination, and anomalous collateral or anastomotic vessels [1,2]. The European Society of Cardiology Working Group has proposed a revised classification, adapting the Angelini framework [3]. This modified approach divides CAAs into three principal domains: anomalies of coronary connection, anomalies of intrinsic anatomy, and anomalies involving myocardial interaction of the coronary arteries [3]. Alternatively, Gentile et al. have offered a more simplified definition, categorizing CAAs broadly as congenital disorders affecting the origin, course, and termination of the coronary arteries [4].

The reported prevalence of CAAs in early angiographic studies ranges from approximately 0.3% to 1.5% [5,6]. However, this prevalence varies widely depending on the diagnostic criteria employed [2]. These anomalies include a wide spectrum of anatomical variants, such as anomalous origin from the opposite sinus, interarterial course, single coronary artery, myocardial bridging, and coronary artery fistulas [1,2,5]. The most common abnormality that was often identified is myocardial bridging [7,8].

Although relatively rare, their clinical significance cannot be underestimated, particularly in relation to myocardial ischemia, arrhythmias, and sudden cardiac death in young adults and athletes [1,9-11]. In many cases, CAAs remain asymptomatic and are detected incidentally during coronary imaging, yet some variants carry a high risk of adverse outcomes and require prompt recognition and management [10].

While international studies have provided valuable insights into the prevalence and anatomical patterns of coronary artery anomalies (CAAs) across diverse populations, there remains a significant gap in national epidemiological data. To the best of our knowledge, there are no published studies specifically addressing the prevalence of CAAs in the United Arab Emirates (UAE) and no data currently available for the Emirate of Dubai. This absence of national-specific epidemiological information highlights a critical gap in the current cardiovascular literature and underscores the need for localized research to better understand the prevalence, clinical implications, and management strategies for CAAs within this population.

## Materials and methods

Study aims and objectives

This study aimed to determine the prevalence of coronary artery anomalies among patients undergoing coronary imaging in Dubai and to analyze the demographic and clinical characteristics associated with these anomalies. It was conducted at a major tertiary care center in Dubai to evaluate the prevalence and anatomical patterns of CAAs. Coronary angiograms were reviewed in the catheterization laboratory at Rashid Hospital, where coronary anatomy was assessed. Clinical presentations and left ventricular systolic function data were extracted from patients’ electronic medical records. By filling a gap in regional data, the findings will contribute to the growing body of literature on CAAs, particularly within the context of the United Arab Emirates, where data remain limited.

Study design and population

A retrospective observational study was conducted at the catheterization laboratory of Rashid Hospital, Dubai, United Arab Emirates. The analysis included all coronary angiography procedures performed between January 1, 2017, and December 31, 2023. All patients who underwent coronary angiography during this period, irrespective of clinical indication, were included in the study to evaluate the prevalence and morphological characteristics of coronary artery anomalies. The study protocol was approved by the Mohammed Bin Rashid University of Medicine and Health Sciences (MBRU) Institutional Review Board (IRB), and the need for informed consent was waived due to the retrospective nature of the analysis. Ethical approval for this study was obtained from the Dubai Scientific Research Ethics Committee (DSREC) of Dubai Health Authority, with reference number DSREC-SR-07/2024_04.

Data collection

All coronary angiograms were independently reviewed by two interventional cardiologists to identify CAAs, utilizing established simplified diagnostic criteria [4]. In the event of differing opinions, a senior interventional cardiologist was consulted to achieve consensus. The following data were extracted from patient records: clinical presentation, demographics, date of angiography, type of anomaly, culprit artery, and in-hospital outcome. Left ventricular systolic function was assessed using left ventricular ejection fraction (LVEF) and categorized as follows: preserved (LVEF: ≥50%), mildly reduced (LVEF: 41%-49%), and reduced (LVEF: ≤40%) [12,13].

Statistical analysis

Continuous variables were expressed as means ± standard deviations (SD), while categorical variables were presented as frequencies and percentages (%). To assess associations between coronary artery anomaly (CAA) subtypes and relevant clinical or echocardiographic parameters, Fisher’s exact chi-square test was applied for categorical comparisons. A 95% confidence interval was maintained throughout, and a P-value of <0.05 was considered statistically significant. All data were analyzed using the Statistical Package for Social Sciences (SPSS), version 23.0 (IBM Corp., Armonk, NY).

## Results

Demographics and temporal distribution

**Figure 1:**
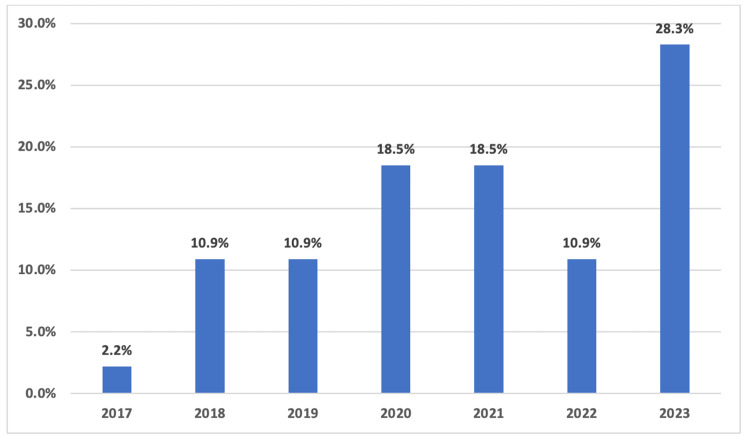
Annual distribution of detected coronary artery anomalies from 2017 to 2023, based on coronary angiography cases performed at Rashid Hospital.

Among the 6,228 coronary arteriograms reviewed, 5,362 (86.1%) were performed on male patients and 866 (13.9%) on female patients. Coronary artery anomalies were identified in 92 patients, representing a prevalence of 1.48%. Of those with CAAs, 79 (85.9%) were men, and 13 (14.1%) were women. The mean age of patients diagnosed with CAAs was 54.04 ± 11.53 years. The annual distribution of CAA detection over the study period is presented in Figure 1.

Clinical presentation and in-hospital outcomes

Among patients diagnosed with CAAs, the most common clinical presentation was acute myocardial infarction (63.0%), followed by chest pain (27.1%), arrhythmia (4.3%), and heart failure (3.3%) (Table 1). All patients underwent echocardiographic evaluation during their admission. Left ventricular ejection fraction was preserved in the majority (67%). Most patients were discharged alive (97.8%), and the in-hospital mortality rate was 2.2% (Table 1).

**Table 1:** Clinical presentation and in-hospital outcomes of patients with coronary artery anomalies. Data are presented as frequency (n) and percentage (%). LVEF: left ventricular ejection fraction

Clinical characteristics	Coronary anomalies (n = 92)
Clinical presentation, n (%)	
Acute myocardial infarction	58 (63.0)
Chronic angina	15 (16.3)
Atypical chest pain	10 (10.9)
Heart failure	3 (3.3)
Arrhythmia	4 (4.3)
Others	2 (2.2)
LVEF categories, n (%)	
Preserved LVEF (≥50%)	61 (67.0)
Mildly reduced LVEF (41%-49%)	15 (16.5)
Reduced LVEF (≤40%)	15 (16.5)
In-hospital outcome, n (%)	
Alive	90 (97.8)
Died	2 (2.2)

Types of coronary artery anomalies

Among the 92 patients diagnosed with CAAs, myocardial bridging was the most frequently observed anomaly, present in 48 patients (52.2%). Anomalies of coronary artery origin were identified in 42 patients (45.7%), making them the second most common anomaly. Coronary artery fistulas were rare, detected in only two patients (2.2%). These findings highlight the predominance of myocardial bridging and origin anomalies within this patient population (Table 2).

**Table 2:** The distribution of coronary artery anomalies among the study population. Data are presented as frequency (n) and percentage (%). LAD, left anterior descending artery; LCX, left circumflex artery; LCA, left coronary artery; RCA, right coronary artery

Type of anomaly	Coronary anomalies (n = 92)	Angiographic prevalence (n = 6,228)
Anomalies of the origin, n (%)	42 (45.7)	42 (0.76)
Separate origin of LAD and LCX from the left sinus of Valsalva (SV)	9 (9.8)	9 (0.14)
LCA from the right sinus of Valsalva	5 (5.4)	5 (0.08)
Left descending artery from the RCA	1 (1.1)	1 (0.02)
LCX from the right sinus of Valsalva	7 (7.6)	7 (0.11)
LCX from the RCA	3 (3.3)	3 (0.05)
RCA from the left sinus of Valsalva	17 (18.5)	17 (0.27)
Anomalies of the course, n (%)	48 (52.2)	48 (0.77)
Myocardial bridging	48 (52.2)	48 (0.77)
Anomalies of the termination, n (%)	2 (2.2)	2 (0.03)
Coronary arterial fistula	2 (2.2)	2 (0.03)

Subtypes of CAAs and relevant clinical or echocardiographic parameters

The clinical presentation varied notably between CAA subtypes. Patients with anomalies of the origin were more likely to present with acute myocardial infarction (73.8%), whereas those with anomalies of the course more commonly reported anginal chest pain (37.5%). These differences approached statistical significance (P = 0.086 for both comparisons), suggesting a trend that may reflect differing hemodynamic or ischemic implications based on anomaly type, as demonstrated in Table 3. A trend toward preserved systolic function was observed in patients with coronary course anomalies, with 72.9% maintaining normal LVEF, compared to those with origin-related anomalies. However, this difference did not reach statistical significance (P = 0.205), as summarized in Table 3.

**Table 3:** Summarizing the distribution of key clinical and echocardiographic parameters within the cohort of patients diagnosed with coronary artery anomalies. Data are presented as frequency (n) and percentage (%). Fisher’s exact chi-square test was applied for categorical comparisons. CAA, coronary artery anomaly; LVEF, left ventricular ejection fraction; AMI, acute myocardial infarction

	CAA of the origin (n = 42)	CAA of the course (n = 48)	CAA of the termination (n = 2)	Value	P-value
Presentation				7.423	0.086
AMI	31 (73.8)	26 (54.2)	1 (50.0)		
Chest pain	6 (14.3)	18 (37.5)	1 (50.0)		
Others	5 (11.9)	4 (8.3)	0 (0.0)		
LVEF				5.684	0.205
Preserved	25 (59.5)	35 (72.9)	2 (100.0)		
Mild reduced	6 (14.3)	9 (18.8)	0 (0.0)		
Reduced	11 (26.2)	4 (8.3)	0 (0.0)		
Outcomes				3.408	0.247
Alive	48 (100.0)	40 (95.2)	2 (100.0)		
Dead	0 (0.0)	2 (4.8)	0 (0.0)		

## Discussion

This study found a CAA prevalence of 1.48%, a figure that aligns with previous early angiographic reports, thereby reinforcing the reliability and validity of our findings [5,6,14]. However, global data indicate a broader range of prevalence estimates, largely influenced by diagnostic modalities and population differences. For instance, a prospective study employing strict diagnostic criteria reported a CAA prevalence as high as 5.6% [2]. Similarly, a recent angiographic study conducted in India found a prevalence of 4.12%, further underscoring the variability in reported rates across different geographic and clinical settings [15]. In contrast, studies from the Gulf region of the Middle East have reported a lower prevalence of CAAs, ranging from 1.0% to 1.3%, based on cardiac computed tomography (CT) angiography [16-18], reflecting rates similar to early angiographic series [5,14].

Myocardial bridging emerged as the most frequently observed coronary artery anomaly in our cohort, accounting for 52.2% of all identified anomalies, with an angiographic prevalence of 0.77%. This observation aligns with previous angiographic studies, which have reported a prevalence of approximately 16.1% [19]. The higher rate observed in our cohort may reflect improved detection techniques or population-specific anatomical variations. However, markedly higher detection rates, ranging from 15% to 86%, have been described in autopsy studies, likely attributable to the superior sensitivity of postmortem examination in detecting intramyocardial segments [20]. Although often considered benign, myocardial bridging has been associated with angina, arrhythmias, and, in rare cases, myocardial infarction, particularly when involving the left anterior descending artery [21,22].

In contrast, anomalies involving the origin of coronary arteries are less frequent but carry greater clinical significance [9,11]. These anomalies are well-documented risk factors for myocardial ischemia, syncope, and sudden cardiac death, especially in cases where a coronary artery arises from the opposite sinus of Valsalva [9,23]. The early identification of such anomalies is essential for risk stratification and appropriate management, including consideration for surgical correction in high-risk cases. In addition, our analysis revealed that most patients with CAAs presented with acute myocardial infarction (63.0%), reinforcing the clinical relevance of these anomalies [21-23]. Additionally, Alkhulaifi et al. from Qatar highlighted a higher prevalence of malignant coronary anomalies in certain Asian populations, suggesting potential geographic or ethnic predispositions [17].

While CAAs are often considered incidental findings, their presence, particularly when associated with ischemia-prone variants such as anomalous origin or myocardial bridging, can contribute to acute coronary syndromes [21,23]. This underscores the importance of considering CAAs in the differential diagnosis of myocardial infarction, especially in patients without traditional atherosclerotic risk factors or with nonobstructive coronary arteries on angiography. Furthermore, the relatively high proportion of preserved left ventricular systolic function (67%) in this cohort suggests that many anomalies may be hemodynamically tolerated, at least in the acute setting. However, the presence of myocardial infarction and a small but notable in-hospital mortality rate (2.2%) highlight that CAAs can carry significant short-term risks, particularly if not recognized promptly.

Limitations

This study has several limitations. First, its retrospective, single-center design may limit the generalizability of the findings to other populations or healthcare settings. Second, the reliance on conventional coronary angiography may have led to the underdiagnosis of certain anomalies that are better characterized with advanced imaging modalities such as cardiac CT angiography or cardiac MRI. Third, the absence of long-term follow-up data restricts our ability to assess the prognostic impact of different CAA subtypes on clinical outcomes beyond hospital discharge.

## Conclusions

In summary, this study confirms that coronary artery anomalies are relatively rare among patients undergoing coronary angiography at Rashid Hospital, with myocardial bridging emerging as the most common anomaly. The observed associations between anomaly type, clinical presentation, and left ventricular systolic function highlight the need for thorough diagnostic assessment in patients with suspected or incidental CAAs.

These findings contribute to the regional literature and support the need for further research into the clinical impact and therapeutic approaches for CAAs. Future multicenter, prospective studies utilizing multimodality imaging and incorporating long-term clinical follow-up are warranted to better define the prevalence, natural history, and prognostic implications of CAAs. Such efforts would also help refine risk stratification and inform evidence-based management strategies tailored to specific anomaly types.
